# The Future of Breast Cancer Diagnosis in Japan with AI and Ultrasonography

**DOI:** 10.31662/jmaj.2024-0183

**Published:** 2024-09-27

**Authors:** Tomoyuki Fujioka, Jitsuro Tsukada, Tetsu Hayashida, Emi Yamaga, Hiroko Tsukada, Kazunori Kubota, Ukihide Tateishi

**Affiliations:** 1 Department of Diagnostic Radiology, Tokyo Medical and Dental University, Tokyo, Japan; 2 Department of Radiology, Keio University School of Medicine, Tokyo, Japan; 3 Department of Surgery, Keio University School of Medicine, Tokyo, Japan; 4 Department of Breast Surgery, School of Medicine, Tokyo Women’s Medical University, Tokyo, Japan; 5 Department of Radiology, Dokkyo Medical University Saitama Medical Center, Koshigaya, Japan

**Keywords:** Breast Cancer, Mammography, Ultrasonography, Artificial Intelligence, Machine Learning, Deep Learning, Computer-Aided Detection, Computer-Aided Diagnosis

## Abstract

In Japan, mammography is commonly used for breast cancer screening. However, the mortality rate has not decreased, possibly due to the low screening uptake and the high prevalence of dense breast tissue among Japanese women, which reduces mammography’s effectiveness. A recent prospective study in Japan, J-START, demonstrated that combining mammography with ultrasonography increases detection rates and reduces the incidence of interval cancers, highlighting the significance of ultrasound examinations.

Artificial Intelligence (AI) technologies, particularly in machine learning and deep learning, offer promising solutions to enhance the accuracy and efficiency of breast ultrasound diagnostics. This review explores AI’s current capabilities in breast ultrasound imaging, emphasizing key advancements in breast lesion detection and diagnosis. Additionally, we introduce AI-based breast ultrasound diagnostic support programs approved by the Pharmaceuticals and Medical Devices Agency, which include programs for detecting lesion candidate regions and diagnosing the necessity of further examination based on detected lesion candidates. These AI tools are expected to improve diagnostic accuracy and efficiency.

While AI holds significant promise, several challenges remain. It is essential for physicians to oversee its use responsibly, as there are concerns regarding patient acceptance and environmental impact. This review underscores the revolutionary potential of AI in breast cancer diagnostics and emphasizes the importance of ongoing research and development to overcome existing limitations.

## Introduction

In Japan, mammography is commonly used for breast cancer screening; however, the mortality rate of breast cancer has not decreased ^(1)^. One reason for this is the low screening uptake. The low participation rate is partly due to the pain associated with mammography and the risk of radiation exposure. Furthermore, compared to Western populations, a higher proportion of Japanese women have dense breast tissue, reducing the breast cancer detection rate through mammography ^(2), (3)^. Consequently, mammographic screening alone may not always be effective in Japan.

Amidst this context, a recent prospective study called J-START was conducted in Japan. This trial demonstrated that adding ultrasonography to mammography increases detection rates and reduces the incidence of interval cancers ^(4)^. Further analysis revealed that the combined approach is effective not only for women with dense breasts, where mammography alone is less effective but also for those with non-dense breasts ^(5)^. This finding highlights the significant role of ultrasonography in breast cancer screening.

Breast ultrasound is an inexpensive, widely used, and simple examination method, but it has issues such as operator dependency, poor reproducibility, and a shortage of skilled operators ^(6), (7)^. Recently, AI technologies have gained significant attention in the medical field, with various beneficial research outcomes reported for different modalities, organs, and diseases, leading to their clinical application ^(8), (9), (10), (11), (12), (13), (14), (15), (16), (17), (18), (19), (20)^.

AI technologies are reportedly useful in breast cancer imaging, potentially overcoming the weaknesses of breast ultrasound examinations and making screenings more effective ^(21), (22), (23), (24), (25)^. This review explores the fundamental knowledge of AI, focusing on breast ultrasound imaging, and examines AI’s current capabilities in this domain and future expectations.

## What Is AI?

AI refers to technologies that mimic human intelligence, enabling the automated and efficient performance of tasks such as data analysis, pattern recognition, decision-making, and prediction. In medicine, AI applications include diagnostic support, image analysis, and treatment planning optimization ^(26)^.

**Figure 1.**
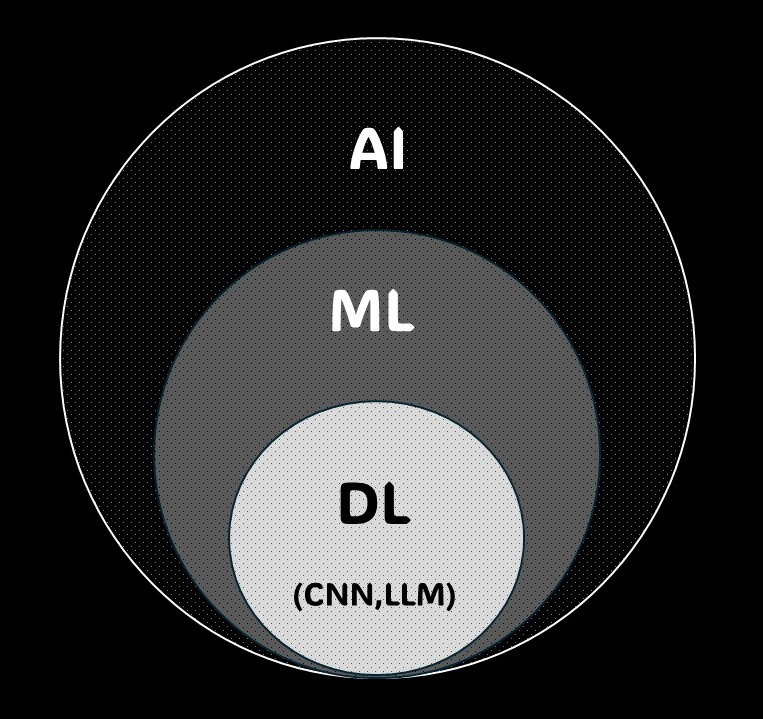
Diagram Showing the Relationships Among AI, ML, and DL Artificial intelligence (AI) is a broad concept that encompasses machine learning (ML), and within ML, there is deep learning (DL). Deep learning has several models, including convolutional neural networks (CNNs) and large language models (LLMs).

Machine learning is a subfield of AI where computers learn from data, improving performance with experience (Figure 1). It involves building models using large datasets to make appropriate predictions or classifications for new data. For instance, algorithms that detect abnormalities in medical images are examples of machine learning ^(26), (27)^.

Deep learning, a form of machine learning, uses artificial neural networks to learn data patterns. With multi-layer network structures, it can extract features from complex data, achieving high performance in tasks like image recognition, speech recognition, and natural language processing. In medical image analysis, deep learning significantly contributes to detecting abnormalities and improving diagnostic accuracy ^(28), (29)^.

Convolutional neural networks (CNNs), a form of deep learning, excel in image data analysis. CNNs use filters to capture spatial features in images by detecting patterns and structures. This technology is highly effective in medical image analysis, including automated detection and classification of abnormalities in breast ultrasonography ^(29)^.

Large language models (LLMs) are AI models used in natural language processing, learning from vast text datasets to understand and generate human language. ChatGPT is one example, and it can answer diverse questions and summarize documents. LLMs have various medical applications, such as automatically analyzing medical records, summarizing documents, and responding to patient inquiries ^(30)^.

## The History of AI Booms and Applications in Medical Imaging

AI has evolved rapidly over the past few decades, playing a crucial role in various fields, including medical imaging diagnosis. There have been three major AI booms, each bringing different technological innovations and applications ^(31), (32)^.

The first AI boom occurred from the late 1950s to the early 1970s, laying the foundation for AI research. During this period, John McCarthy coined the term “artificial intelligence ^(33)^”. Early AI systems targeted tasks like chess and puzzle-solving, developing basic logic algorithms. However, practical applications were limited due to the performance and data constraints of the computers at the time.

The second AI boom focused on expert systems from the 1980s to the mid-1990s. These systems used specialized knowledge to provide practical medical diagnosis and financial analysis applications. Widely researched rule-based systems faced challenges such inflexibility and management difficulties. Nonetheless, this period expanded AI’s application range and laid the groundwork for the next boom ^(32)^.

The third AI boom began in the early 2000s, with significant advancements in machine learning and deep learning technologies. With the internet’s proliferation and computational power improvements, handling large datasets became feasible, leading to the development of highly accurate AI models ^(31)^.

**Figure 2.**
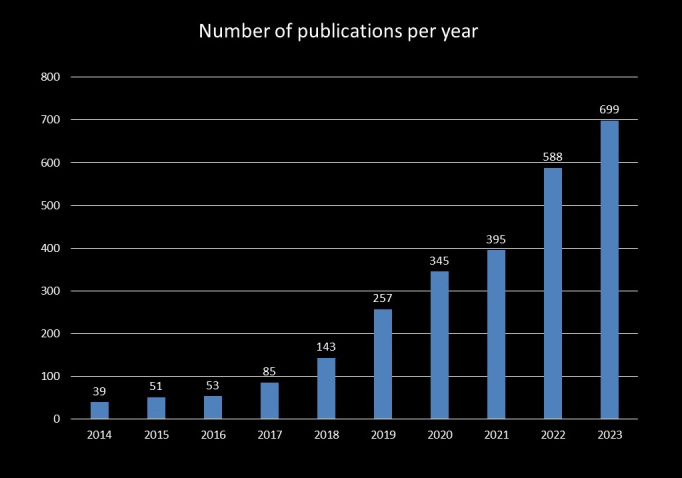
Annual Number of Publications on AI in Breast Ultrasound This figure shows the annual number of publications on Artificial intelligence (AI) in breast ultrasound from 2014 to 2023. The data, extracted from PubMed using a specific query, indicates a steady number of publications from 2014 to 2016, followed by a significant increase starting in 2017. Publications exceeded 100 annually since 2018, surpassing 300 in 2020, and reaching 699 in 2023.

Breast ultrasonography research also advanced during the third AI boom, increasing the number of related publications ^(34)^. As of 25 June 2024, we extracted studies related to image diagnostics and AI in breast ultrasound using the following PubMed search query: (‘artificial intelligence’ OR ‘machine learning’ OR ‘deep learning’) AND (‘US’) AND (‘breast’). The annual number of publications from 2014 to 2016 remained constant, but there was a significant increase from 2017 to 2020, particularly in deep learning research. Since 2018, the annual number of publications on AI in breast ultrasound has exceeded 100, surpassing 300 in 2020 and reaching 699 in 2023. Many of these studies focus on image classification, object detection, segmentation, and image reconstruction of breast lesions (Figure 2).

## CAD in Diagnostic Image

Computer-aided detection/diagnosis (CAD) systems are computer technologies that detect abnormalities or lesions in medical images, support radiologists, and improve detection rates. CAD has two main functions: detection (CADe) and diagnosis (CADx). CADe aims to detect abnormalities or lesions within medical images by scanning the entire image and marking suspicious areas, helping radiologists identify small lesions and abnormalities that may be easily missed. For instance, CADe is used in mammography for detecting microcalcifications and masses in breast cancer screening. CADx evaluates the nature of detected abnormalities or lesions, aiding in diagnosis. This function analyzes the characteristics of detected areas to determine if they are benign or malignant or to assess other features. For example, CADx can differentiate between benign and malignant lung nodules ^(35)^.

Integrating CAD systems can significantly enhance diagnostic efficiency. By prioritizing cases that need closer attention, CAD allows specialists to focus on the most pertinent images, improving overall workflow and diagnostic accuracy ^(36)^.

## How to Use AI for Diagnostic Image

**Figure 3.**
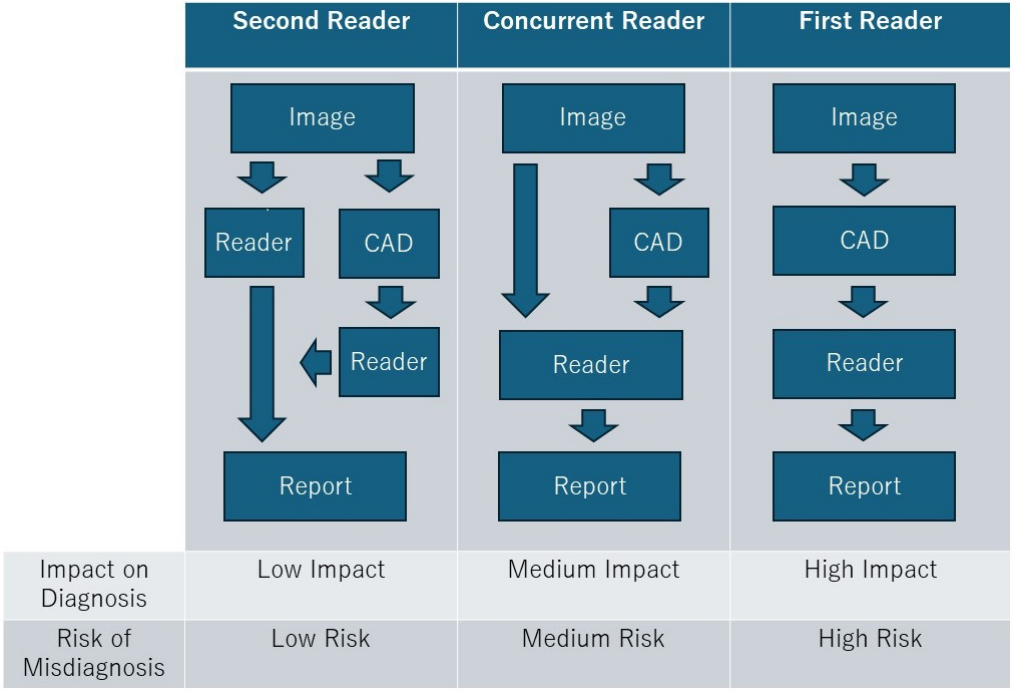
Approaches to CAD-Assisted Image Reading Three artificial intelligence (AI) reading approaches exist: second-reader, concurrent-reader, and first-reader. The second-reader method involves initial radiologist reading followed by AI reanalysis. The concurrent-reader method uses real-time AI analysis during reading. The first-reader method has AI analyze images first, then the radiologist reviews AI-identified candidates.

There are three approaches to AI reading: second-reader, concurrent-reader, and first-reader (Figure 3) ^(31), (35)^. In the second-reader approach, the radiologist reads the images without AI results, followed by a second review with AI reanalysis for double-checking. Many currently approved AI products adopt this approach. This method supplements the radiologist’s reading, reduces missed detections, and improves diagnostic accuracy, especially in important examinations and diagnoses.

In the concurrent-reader approach, the radiologist refers to AI analysis results in real-time while reading the images, which enhances diagnostic accuracy and efficiency. By immediately incorporating AI results, this method enables rapid diagnosis, suitable for emergency medical situations and intraoperative image analysis.

The first-reader approach involves AI analyzing the images first, with the radiologist then diagnosing based on the AI-identified candidates. Although this method could significantly improve reading efficiency, it requires high analytical performance and faces regulatory hurdles, making implementation challenging.

When using AI in image diagnosis, it is essential to understand the best approach to maximize the AI’s utility.

## Current Status of Medical Device Programs

In Japan, the development and approval of AI-based medical device programs are progressing, with increasing approvals from the Pharmaceuticals and Medical Devices Agency (PMDA). These AI medical device programs are crucial diagnostic support tools in clinical settings. Table 1 shows the AI medical device programs currently approved by the PMDA for X-ray, CT, MRI, and ultrasound (Table 1). Since 2019, various programs have been approved annually, with the most for CT and the second for X-ray. These programs mainly aim to detect lung nodules and support pneumonia detection. In breast image diagnosis, specific programs have been approved.

**Table 1.** AI Medical Device Programs Currently Approved by the PMDA for X-ray, CT, MRI, and Ultrasound.

Approval Date	Product Name	Company	Modality	Description
2019.09	Medical Image Analysis Software EIRL aneurysm	LPixel	MRI	Detects candidate points similar to arterial aneurysmal deformation.
2019.12	Similar Image Case Search Software FS-CM687	Fujifilm	CT	Analyzes target areas (lung nodules, diffuse diseases, liver tumors) and supports the search for similar images.
2020.05	Lung Nodule Detection Program FS-AI688	Fujifilm	CT	Detects candidates with lung nodule-like shadows.
2020.06	COVID-19 Pneumonia Image Analysis AI Program InferRead CT Pneumonia	CES Descartes	CT	Indicates the possibility of COVID-19 pneumonia based on image findings and marks related areas.
2020.06	AI-Rad Companion	Siemens Healthcare	CT	Provides functions such as lung nodule detection and measurement/display of lung parenchyma.
2020.06	COVID-19 Pneumonia Image Analysis Program Ali-M3	MIC Medical	CT	Indicates possible COVID-19 pneumonia based on image findings and marks related areas.
2020.08	Medical Image Analysis Software EIRL X-Ray Long Nodule	LPixel	X-ray	Detects candidate areas suspected of having lung nodules.
2020.11	Breast Cancer Diagnosis Support Program RN-CES Descartes	CES Descartes	Ultrasound	Detects lesion candidate areas.
2021.05	COVID-19 Pneumonia Image Analysis Program FS-AI693	Fujifilm	CT	Indicates possible COVID-19 pneumonia based on image findings and marks related areas.
2021.07	Chest X-ray Lesion Detection (CAD) Program LU-AI689	Fujifilm	X-ray	Detects candidate areas of lung nodules, tumor shadows, infiltrative shadows, and pneumothorax.
2021.09	Rib Fracture Detection Program FS-AI691	Fujifilm	CT	Marks rib fracture or suspected fracture areas.
2021.10	Image Diagnosis Support Software KDSS-XR-AI-101	Konica Minolta	X-ray	Supports detection of nodular shadows and infiltrative shadows.
2021.12	Chest X-ray Pneumonia Detection Engine DoctorNet JLK-CRP	DoctorNet	X-ray	Displays possible imaging findings seen in infectious pneumonia.
2021.12	HOPE LifeMark-CAD Pneumonia Image Analysis Support Program for COVID-19	Fujitsu Japan	CT	Indicates possible COVID-19 pneumonia based on image findings and marks related areas.
2022.06	COVID-19 Pneumonia Analysis Software SCO-PA01	Canon Medical Systems	CT	Indicates possible COVID-19 pneumonia based on image findings and marks related areas.
2022.12	Medical Image Analysis Software EIRL Chest XR	LPixel	X-ray	Detects candidate areas suspected of having abnormal shadows (nodules, consolidations, atelectasis, interstitial shadows).
2023.11	Fibrosis-Associated Interstitial Lung Disease Detection Support Program BMAX	Cosmotec	X-ray	Detects findings associated with fibrosis in interstitial lung disease and present their confidence scores.
2024.05	Smart Opinion METIS Eye	Smart Opinion	Ultrasound	Detects candidate areas of suspected lesions and determines whether detailed examination is necessary.

**Figure 4.**
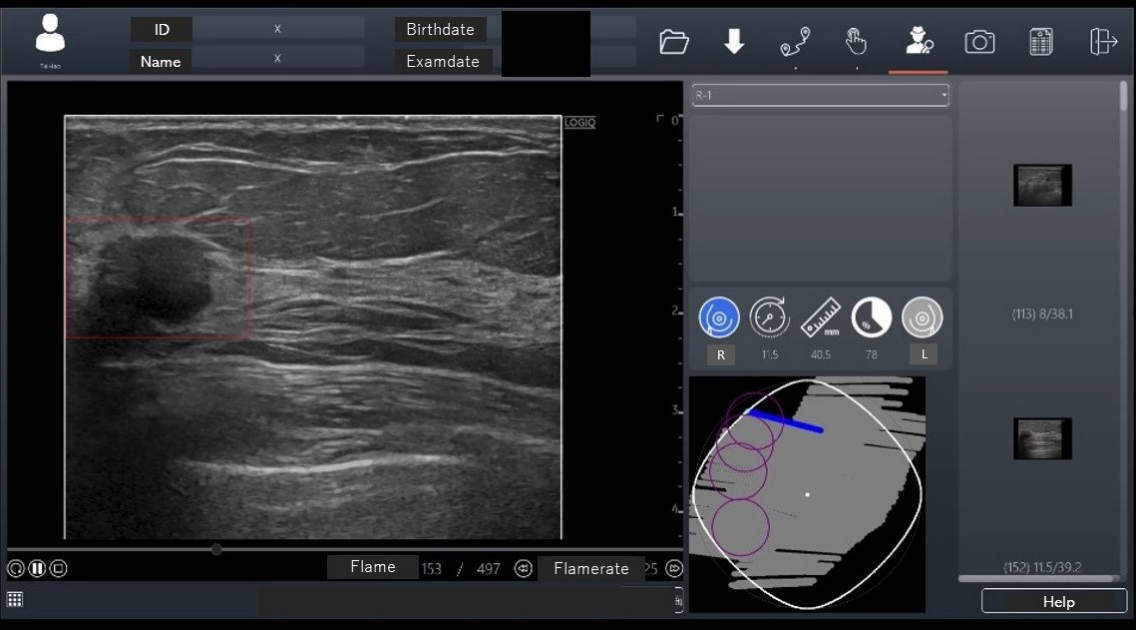
Detection of Breast Lesions Using CADe in Ultrasound In November 2020, a breast cancer detection support program was approved for the ultrasound modality in Japan for the first time. Artificial intelligence (AI) has two functions: it performs shape analysis during the examination, displaying suspected breast cancer areas as regions of interest on the image screen. The probe position is also shown on a route map screen.

In November 2020, an AI breast cancer diagnosis support program, RN-Descartes (CES Descartes, JAPAN), was approved for ultrasound modality. This program detects lesion candidate regions from ultrasound images (CADe) and, developed in Taiwan, is sold under the name BR-FHUS Smart System™ (TaiHao Medical, Taiwan) outside Japan ^(37)^. The program used 88 image sets from 45 cases (each with about 700 B-mode video frames) as training images, using a modified YOLOv3-tiny model for deep learning to train real-time lesion detection. The AI has two functions: it performs shape analysis during the examination, displaying suspected breast cancer areas as regions of interest on the image screen. The probe position is also shown on a route map screen (Figure 4). Eighteen readers evaluated 52 test image sets with and without CADe. The image set’s area under the curve (AUC) was 0.7726 with CADe and 0.6304 without CADe, a significant increase of 0.1422 (p < 0.0001). The sensitivity per case was higher with CADe (95.4%) than without CADe (83.7%). The specificity of suspected breast cancer cases was higher with CADe (86.6%) than without CADe (65.7%). The false-positive count per case was lower with CADe (0.22) than without CADe (0.43). Based on these results, this CADe system received PMDA approval in Japan for the first time (approval number: 30200BZX00379000).

In May 2023, Smart Opinion METIS Eye (Smart Opinion, JAPAN) was approved as medical device software for AI-assisted diagnosis of breast ultrasound images in Japan (approval number: 30600BZX00086000). This software employs AI technology based on CNN. It was developed using annotated breast ultrasound images provided by Keio University and its collaborative research institutions. A notable feature of this software is its use of the BI-RADS classification, globally recognized as a diagnostic standard for breast ultrasound findings ^(38)^, to instantly determine whether detailed examination is required and distinguish between benign and malignant lesions.

Evaluations using the BI-RADS classification have reported a high negative predictive value of over 99% ^(39), (40)^, and the likelihood of malignancy is very low for evaluations of BI-RADS category 3 or lower. Therefore, Smart Opinion METIS Eye is designed as a screening tool to distinguish between BI-RADS 3 or lower (no detailed examination required) and BI-RADS 4a or higher (detailed examination required), based on the BI-RADS classification.

The training data was collected in cooperation with collaborative research institutions, using ultrasound images of cases histologically diagnosed as benign or malignant via needle biopsy and cases judged to be clinically benign upon re-examination after more than six months of follow-up. Annotations were made on 8,670 lesions within breast ultrasound images, with a dataset comprising 4,028 images and 5,014 lesions for training and 3,166 images and 3,656 lesions for testing. The AI diagnostic system was trained using these data.

The ROC curve for AI’s judgments demonstrated a high AUC of 0.95, indicating reliable discriminatory power, with a sensitivity of 91.2% and a specificity of 90.7%. The Japanese Breast Cancer Screening Accuracy Management Central Organization requires a sensitivity and specificity of over 80% for ultrasound-certified physicians, which is considered a passing mark. The AI system met these standards. Additionally, in a test involving 20 surgeons with 5-8 years of experience using a subset of images for BI-RADS classification, the median sensitivity was 67.1%, and the median specificity was 81.4%, with the highest sensitivity at 84.2% and specificity at 90.9%. In contrast, AI achieved 100% sensitivity and 90.9% specificity, demonstrating that AI outperformed clinicians in sensitivity and specificity (p < 0.001) ^(41)^.

## Expectations for Medical Device Programs

Western countries have approved several AI medical device programs for mammography ^(42)^, but the PMDA in Japan has approved none. Different regulations and market demands may contribute to this situation, but there is a growing expectation for developing and approving mammography AI programs in Japan. However, introducing such AI from overseas, particularly Western countries, requires caution. A previous study tested a mammography AI program developed with Western data on 340 Japanese cases and found that while human readers had an AUC of 0.816, the AI achieved an AUC of 0.706, indicating limited effectiveness ^(43)^. Therefore, careful consideration is needed when importing AI from abroad.

**Figure 5.**
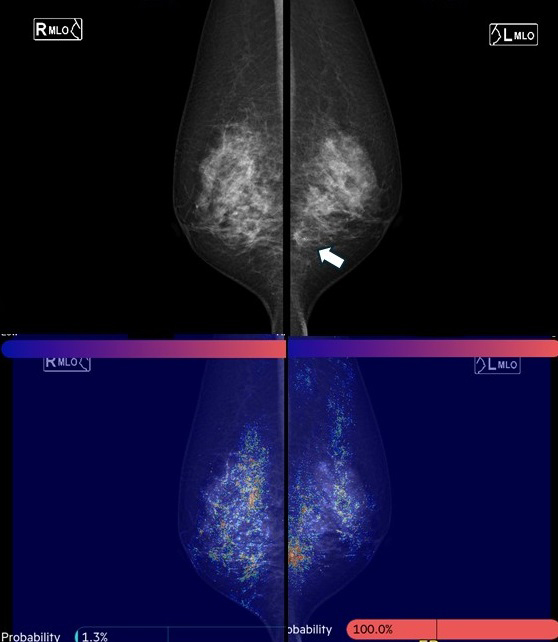
AI Analysis of Mammography Images A mass is observed in the lower region of the left mediolateral oblique view (→). Artificial intelligence (AI) quantifies the probability of malignancy and highlights areas of interest on a heat map when mammography images are uploaded. This case was diagnosed as left breast cancer. The AI correctly identified it as breast cancer.

Since 2019, the authors have collaborated with the National University of Singapore to develop a mammography AI for Asian women. Using CNNs and graph convolutional networks, this AI model quantifies the probability of malignancy and highlights areas of interest on a heat map when mammography images are uploaded (Figure 5). The model demonstrated a high AUC of 0.902 for breast cancer detection. Assuming a cancer miss rate of 3% (the current standard is 3-18%), up to 38% of normal mammograms could be safely excluded from human review. This mammography AI, named FxMammo (FathomX Pte Ltd, Singapore), was approved in Singapore in 2022 and Malaysia in 2023. Research on mammography AI is one of the most active fields, and there is significant anticipation for its use in Japan.

Automated breast ultrasound (ABUS) offers the advantage of high reproducibility and reduced operator dependency due to its capability for 3D volume scanning ^(44)^. However, ABUS also presents challenges, such as the need for extensive data processing, which can extend reading times, and a limited number of devices, leading to fewer experienced readers. Integrating AI programs with ABUS can enhance its utility as a diagnostic tool by improving accuracy, reducing reading time, and alleviating stress.

**Figure 6.**
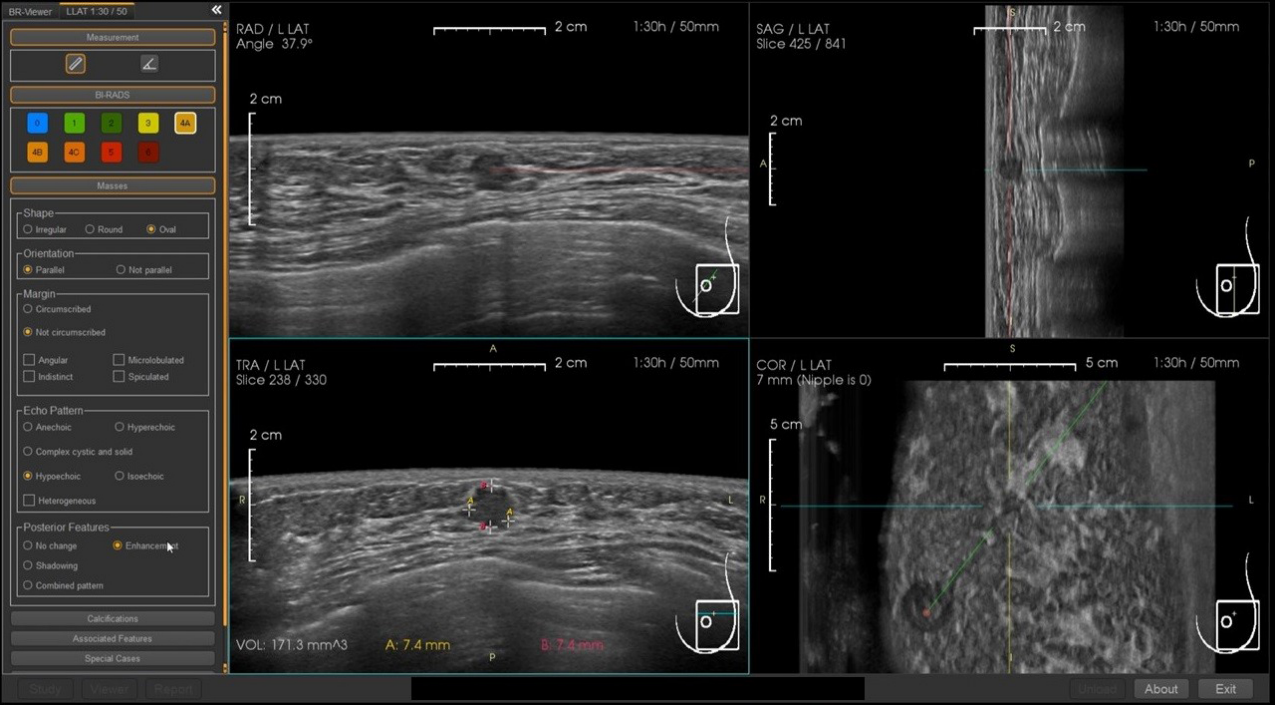
Lesion Identification and Evaluation with an AI System for ABUS An artificial intelligence (AI) system for Automated Breast Ultrasound (ABUS) can identify lesion candidates and provide precise locations and sizes of lesions. Additionally, it evaluates the shapes and findings of lesions based on BI-RADS (Breast Imaging Reporting and Data System), aiding in diagnosing BI-RADS categories. This figure illustrates how the system has the potential to enhance diagnostic accuracy and efficiency, complementing radiological reading.

**Figure 7.**
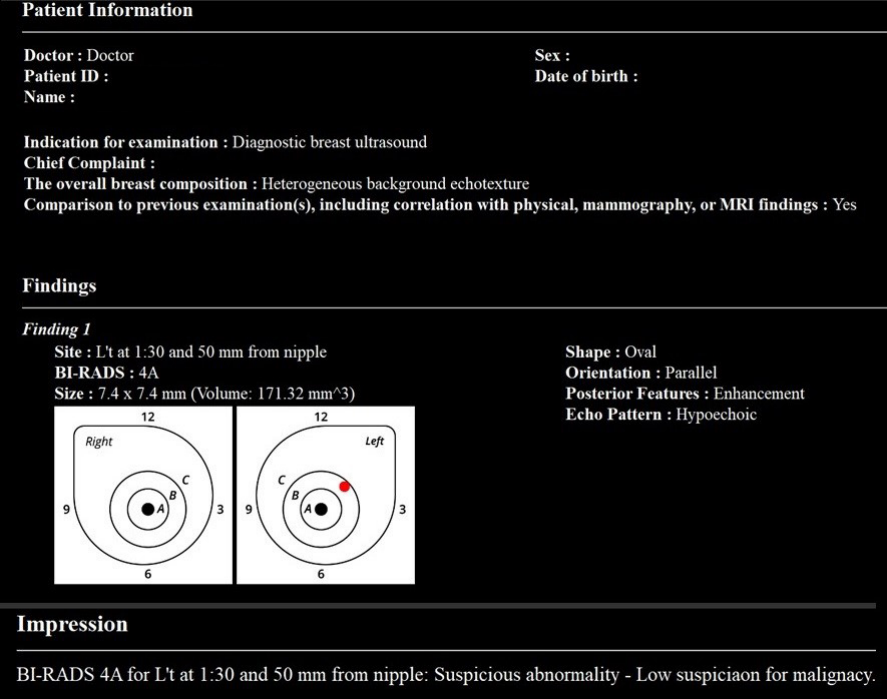
Automatic Selection of Key Images and Report Generation with an AI System for ABUS An artificial intelligence system for Automated Breast Ultrasound (ABUS) features automatic selection of key images and generation of reading reports, which can significantly reduce the time required for reading and report preparation. This figure demonstrates the potential capability of the system to streamline the diagnostic process, making it more efficient for radiological reading.

Although not yet approved in Japan, the BR-Viewer™ (TaiHao Medical, Taiwan) is an AI system compatible with ABUS. This system identifies lesion candidates and provides precise locations and sizes of lesions. Additionally, the shapes and findings of lesions are evaluated based on BI-RADS, aiding in the diagnosis of BI-RADS categories (Figure 6). This complements the physician’s diagnosis, enhancing diagnostic accuracy and efficiency. It also features automatic selection of key images and generation of reading reports, significantly reducing the time required for reading and report preparation (Figure 7).

## Challenges of AI Programs

AI programs offer many benefits, but there are several challenges to consider. Firstly, the ultimate responsibility lies with the physician, who must use AI knowledgeably and interpret AI-provided diagnostic results accurately. While AI is effective, physicians should lead in image management and diagnosis.

It is also necessary to clearly explain to patients how AI is used in diagnosis, as they may prefer explanations and diagnoses from physicians rather than AI. Furthermore, the environmental impact of AI technology usage is a concern. Large data centers running AI consume significant amounts of electricity, potentially affecting the environment. Therefore, sustainable methods for utilizing AI technology are also important ^(45)^. The issue of personal information protection is also important. Medical images contain patient information; when analyzing them with AI, it is essential to protect personal information. Appropriate measures, such as data anonymization, encryption, and access control, are required to safeguard patient privacy. It is also necessary to ensure transparency regarding handling personal information and to explain to patients how their information will be used.

Moreover, the issue of AI fairness is critical. As AI integration in clinical settings progresses, concerns about AI biases and discrimination impacting patient health have arisen. Addressing these concerns requires diverse and representative data and thorough algorithm audits. Cooperation among physicians, AI researchers, developers, policymakers, and patients is essential to ensure equitable AI integration ^(46)^.

Considering these challenges, careful approaches are required to develop and use AI programs. However, if properly managed, AI technology can bring significant advancements to the medical field. Solutions to these challenges are expected with future technological progress.

## Conclusion

Technological advancements have developed increasingly sophisticated AI programs, and more information and achievements should be reported. AI can significantly improve diagnostic accuracy and efficiency in clinical settings, potentially establishing new standards, especially in breast cancer diagnosis. AI technology is crucial for supporting early detection and accurate diagnosis of lesions, improving patient prognosis.

In the future, healthcare professionals should acquire the knowledge and skills to effectively utilize AI, enhancing the quality of medical care through collaboration with AI. AI technology holds great potential for revolutionizing diagnostic and treatment processes in clinical settings, contributing to improved patient care. Such progress will lead to a brighter future for breast cancer diagnosis.

## Article Information

### Conflicts of Interest

None

### Acknowledgement

We used GPT-4 (https://chat.openai.com/) for Japanese-English translation and English proofreading. The authors read, revised, and proofed the generated text.

### Approval by Institutional Review Board (IRB)

Not applicable.

### Disclaimer

Ukihide Tateishi is one of the Editors of JMA Journal and on the journal’s Editorial Staff. He was not involved in the editorial evaluation or decision to accept this article for publication at all.
